# A Placebo-Controlled Trial of Riboflavin for Enhancement of Ultramarathon Recovery

**DOI:** 10.1186/s40798-017-0081-4

**Published:** 2017-03-28

**Authors:** Martin D. Hoffman, Taylor R. Valentino, Kristin J. Stuempfle, Brandon V. Hassid

**Affiliations:** 1 Department of Physical Medicine & Rehabilitation, Department of Veterans Affairs, Northern California Health Care System, Sacramento, CA USA; 2 Ultra Sports Science Foundation, El Dorado Hills, CA USA; 3 Department of Kinesiology, San Francisco State University, San Francisco, CA USA; 4 Health Sciences Department, Gettysburg College, Gettysburg, PA USA; 5 School of Medicine, University of Maryland, Baltimore, MD USA

**Keywords:** Creatine kinase, Muscle fatigue, Muscle pain, Muscle soreness, Running

## Abstract

**Background:**

Riboflavin is known to protect tissue from oxidative damage but, to our knowledge, has not been explored as a means to control exercise-related muscle soreness. This study investigated whether acute ingestion of riboflavin reduces muscle pain and soreness during and after completion of a 161-km ultramarathon and improves functional recovery after the event.

**Methods:**

In this double-blind, placebo-controlled trial, participants of the 2016 161-km Western States Endurance Run were assigned to receive a riboflavin or placebo capsule shortly before the race start and when reaching 90 km. Capsules contained either 100 mg of riboflavin or 95 mg of maltodextrin and 5 mg of 10% ß-carotene. Subjects provided muscle pain and soreness ratings before, during, and immediately after the race and for the 10 subsequent days. Subjects also completed 400-m runs at maximum speed on days 3, 5, and 10 after the race.

**Results:**

For the 32 (18 in the riboflavin group, 14 in the placebo group) race finishers completing the study, muscle pain and soreness ratings during and immediately after the race were found to be significantly lower (*p* = .043) for the riboflavin group. Analysis of the 400-m run times also showed significantly faster (*p* < .05) times for the riboflavin group than the placebo group at post-race days 3 and 5. Both groups showed that muscle pain and soreness had returned to pre-race levels by 5 days after the race and that 400-m run times had returned to pre-race performance levels by 10 days after the race.

**Conclusions:**

This preliminary work suggests that riboflavin supplementation before and during prolonged running might reduce muscle pain and soreness during and at the completion of the exercise and may enhance early functional recovery after the exercise.

## Key Points

- This study provides some evidence that riboflavin supplementation immediately before and during prolonged running may reduce muscle pain and soreness during and at the completion of the run.
- The study also offers some suggestion that riboflavin supplementation immediately before and during prolonged running might enhance functional recovery after the run.
- The findings should be considered preliminary and are based on a relatively small number of subjects but are particularly intriguing and warrant further investigation since the dose and dosing schedule might not have been optimal.

## Background

Post-exercise muscle pain and soreness have been well documented in ultramarathon running [1–8]; however, the precise etiology and pathophysiology remain elusive. Presumably, the process begins with mechanical damage to the muscle and connective tissue; is followed by inflammation, swelling, and the production of free radicals; and culminates in the pain and soreness felt during and after exercise [9–11]. Markers of inflammation and oxidative stress are known to be high following an ultramarathon [5, 7, 12–15].

Numerous pre- and post-exercise interventions using nutritional supplements and dietary strategies have been investigated for prevention or treatment of exercise-related muscle pain and soreness [10, 16]. Since the inflammatory response and free radical production with oxidative stress are likely involved in the mechanism leading to exercise-related muscle soreness, it seems plausible that supplementation with substances having anti-inflammatory or antioxidant properties may be an effective means of controlling such soreness. While research has provided some general support for the effectiveness of such substances in reducing exercise-related muscle soreness [10, 16], previous studies specific to ultramarathon running have demonstrated no effect. For instance, 6 weeks of vitamin E and C supplementation was found to have no effect on muscle damage, inflammatory markers, or muscle function recovery after a 50-km trail run [12, 13]. Furthermore, 3 weeks of supplementation with quercetin, another substance with known antioxidant properties, was not found to alter antioxidant capacity or oxidative damage, inflammation, muscle damage, or post-race muscle soreness from a 161-km ultramarathon [6, 15].

A common nutritional supplement that we believe has not been investigated for its effect on exercise-related muscle soreness is vitamin B2 (riboflavin). As for other flavonoids, riboflavin is known to exhibit antioxidant properties and protect tissue from oxidative damage [17–23]. Riboflavin is also important in cell repair and production and for protecting mitochondrial and other enzymes as a mitochondrial enzyme cofactor or cofactor precursor [24]. It is one of eight water-soluble B vitamins found in many foods and must be regularly supplied in the diet as it is not stored by the body [25]. Human trials have largely focused on the efficacy of riboflavin supplementation for migraine prophylaxis with favorable findings [26, 27]. Whether or not it might provide a protective role or enhanced recovery from exercise-induced skeletal muscle damage is unknown.

The purpose of this study was to investigate whether acute ingestion of riboflavin is effective at reducing muscle pain and soreness during and after completion of a 161-km ultramarathon, and in improving functional recovery after the event. The study was performed in association with the 161-km Western States Endurance Run (WSER) since this race is known to induce considerable muscle damage and pain [2, 3, 5–7, 28–32]. Based upon the evidence that riboflavin can protect tissue from oxidative damage and is important in cell repair, we hypothesized that riboflavin would be effective at reducing muscle pain and soreness and improving muscle recovery after this extreme level of exercise.

## Methods

### Study Design and Subjects

This double-blind, placebo-controlled trial was performed at the 2016 WSER, a 161.3-km ultramarathon through the Sierra Nevada Mountains of Northern California. The course is mostly single-track trail with 5500 m of cumulative climb and 7000 m of cumulative descent. Other race details have been provided elsewhere [33–36]. Nearby weather station ambient temperatures during the race ranged from a low of 1 °C at the start to a high of 34 °C. The San Francisco State University Institutional Review Board provided approval for the research with electronic consent obtained during online enrollment and formal consent obtained at race registration. Study participants completing the pre-race data collection were provided a t-shirt, and those completing the entire study were provided a $50 (USD) gift certificate.

Subject recruitment was by electronic notices sent to all race entrants 59 and 19 days before the race. Because of inadequate subject recruitment, additional subjects were recruited during race registration held the day before the race start.

All study participants were met during race registration for formal consenting, additional pre-race data collection, and review of expectations for participation. Subjects were then seen for further intervention before and during the race as outlined below. Immediately after race completion, subjects were escorted 30 m to a tent adjacent to the finish line where post-race data collection and a blood draw were performed. During the subsequent 10 days, the subjects recorded additional information that was then returned to the investigators electronically or by mail. They were sent an email the evening after the race reminding them of the post-race data collection and were provided an electronic copy of the data sheet at that time.

Subjects were asked to avoid any use of pain or nonsteroidal anti-inflammatory drugs (NSAIDs) during the race. Additionally, subjects were asked to avoid use of pain medications or NSAIDs, compression garments, massage, electrical stimulation, and thermal modalities in the 10 days following the race. To check compliance, the post-race data form requested that they provide details about use of pain medications or NSAIDs during or after the race or any of the above interventions during the 10 days after the race. Subjects were also asked which group they thought they were in and why.

### Intervention

Subjects were assigned to the riboflavin or placebo group in an alternating fashion based on the order of arrival to meet with the research team at race registration, with the on-site researchers and subjects being blinded to the group assignment. The subjects were told that they would receive either a placebo or an essential vitamin with flavonoid properties, but no other details of the supplement under study were provided until after data analysis had been completed.

All subjects received a capsule 0.5–1 h before the start of the race with a cup of water and were observed to swallow the capsule. They were also provided and observed to take another capsule at the 90-km aid station. Capsules were prepared by the investigators using riboflavin (Nature’s Way Products, Inc., Green Bay, WI), maltodextrin (Now, Bloomingdale, IL), and 10% ß-carotene powder (BulkSupplements.com, Henderson, NV). The treatment group received capsules containing 100 mg of riboflavin, and the control group received capsules containing 95 mg of maltodextrin and 5 mg of 10% ß-carotene. Because riboflavin can cause urine to appear bright or fluorescent yellow [37] and might allow subjects to suspect they were in the treatment group, the small amount of ß-carotene was added to the placebo, as done in a prior placebo-controlled trial of riboflavin [27], since it can also cause a similar urine color change. The amount of ß-carotene in each capsule was approximately half of that in a single large raw baby carrot [38], so we recognize it was unlikely to cause marked urine discoloration, but it did allow us to legitimately tell the subjects that they might notice a discoloration of the urine regardless of their group assignment. We also recognized that ß-carotene is an antioxidant but felt that it would have no recognizable antioxidant effect at such a low dose.

### Measurements

#### Body Weight

The body weight of each subject was obtained at race registration and immediately after finishing the race using the same scale (Sunbeam Products, Inc., Health o meter, model 349KLX, Boca Raton, FL) placed on a firm, level surface. For each measurement, the runner was clothed in running attire and shoes but had no other items on their body or in their hands.

#### Plasma Creatine Kinase Concentration

Plasma creatine kinase (CK) concentration was determined immediately post-race from a blood sample taken from the antecubital vein, with subjects seated in the upright position. The samples were centrifuged for 10 min at 3400 rpm within 10 min of collection and then stored in a cooler until transported to and analyzed by a clinical laboratory for plasma CK concentrations (Siemens Aktiengesellschaft, Dimension EXL, Munich, Germany).

#### Subjective Measurement of Muscle Pain and Soreness

Runners were asked to rate their perceived lower-body muscle pain and soreness according to a 10-point Likert scale with anchors of 1 (no soreness), 2.5 (dull, vague ache), 4 (slight soreness), 5.5 (more than slight soreness), 7 (sore), 8.5 (very sore), and 10 (unbearably sore). This approach has been previously used at the WSER [2–7] and elsewhere [39], and the values have been found to correlate with plasma CK concentrations [3–5]. Ratings were provided at race registration, during the race at the 48-, 90-, and 126-km checkpoints, at the finish prior to the blood draw, and each morning of the 10 days following the race after being up and moving around for approximately 30 min.

#### Functional Measurement

A 400-m run at maximal speed was used as a functional measurement, which we have previously used successfully [2, 3]. Those subjects enrolled in advance of race registration were asked to perform this test twice on separate days during the 21 days before the race, and all subjects were asked to perform the test on days 3, 5, and 10 after the race. Subjects were sent an electronic reminder the night before each post-race 400-m run. This test is functionally specific for running, but not overwhelmingly long and painful or so short and intense that it would be a high risk for inducing a strain injury. After an adequate warm-up, subjects were instructed to self-time the run, starting from a standing start. Subjects were asked to use the inside lane of a running track, assuring an accurate distance. In the event they did not have access to a track, they were allowed to utilize a level section of road that had been measured to the correct distance. They were asked to use the same site for all pre-race and post-race testing and under conditions without significant wind or other environmental variations.

### Statistical Analyses

Comparison of treatment and control groups (age, sex, finish time, percent change in body mass from registration to immediately post-race, post-race plasma CK concentration, pre-race 400-m run time, average weekly running distance, highest weekly running distance, and longest training run) were made using unpaired *t* tests and the chi-square test. Main outcome variables (muscle pain and soreness rating and 400-m run time) were compared between groups with two-way (group × time) repeated measure analysis of variance (ANOVA) and Bonferroni post-tests. These data were tested for normality with the D’Agostino-Pearson normality test. The 400-m run time data were skewed and successfully normalized before analysis with the reciprocal function (i.e., transformed value = 1/original value). Statistical significance was set at *p* < 0.05.

A priori sample size determination was performed based on muscle pain and soreness ratings from previous research at the WSER [2–4]. Using a level of significance of *p* < 0.05, an expected common SD of 1.5 points, and 80% power, the predicted minimum sample size to determine a meaningful group difference of 1.5 points was 13 per group. Recent research at the WSER has shown attrition rate to be roughly 25–30% [2, 3, 40], so we aimed to recruit a minimum of 17 subjects per group.

## Results

Of the 353 race entries, 44 runners enrolled in the study (22 in each group) and started the race. Of this group, 37 (84.1%, 20 in riboflavin group, 17 in placebo group) finished the race and 32 (18 in riboflavin group, 14 in placebo group) completed data collection. Of the 32 completing data collection, 8 had enrolled in advance of race registration and completed both pre-race 400-m run trials (5 in riboflavin group, 3 in placebo group). Overall race finish rate was 79.3% (280 of 353 starters), which was similar (*p* = .55) to that for the study participants.

Selected characteristics of the subjects completing the study are shown in Table 1. None of the examined characteristics differed between groups, including the post-race plasma CK concentrations. Furthermore, race finish rate (91 and 77% for the riboflavin and placebo groups, respectively) and study completion rate (82 and 64% for the riboflavin and placebo groups, respectively) did not differ statistically (*p* = .4 and *p* = .3, respectively) between groups. One subject in the placebo group failed to receive the capsule at 90 km, and two subjects in the riboflavin group reported emesis within an hour after taking the capsule at 90 km. All other subjects received both doses and were confirmed to not have had emesis during the hour after taking a capsule. Post-race exercise behavior appeared comparable between groups, and during the first 2 days post-race, none of our subjects reported exercise more taxing than running less than 2 km or some walking.

**Table 1:** Selected characteristics of the two study groups

Characteristic	Riboflavin (*n* = 18)	Placebo (*n* = 14)
Age (years)	44 ± 8	46 ± 7
Sex (% men)	83	79
Average weekly running distance (km)^a^	83 ± 20	94 ± 20
Highest week running distance (km)^a^	135 (126–156)	129 (120–142)
Longest training run or race (km)^a^	80 (66–92)	80 (56–97)
Body mass change from registration to post-race (%)	−3.6 ± 2.1	−2.6 ± 2.0
Finish time (hours)	27.26 ± 2.36	27.08 ± 2.88
Used any pain medication during the race (%)	50	36
Used any pain medication in 10 days post-race (%)	33	43
Post-race plasma CK (U/L)	6804 (3536–24,592)	12,819 (7560–46,965)

Data are reported as mean ± SD, a percentage, or median (interquartile range) if the data were skewed for either group

^a^Distances are during the 3 months prior to the race. No significant group differences were present

**Fig. 1:**
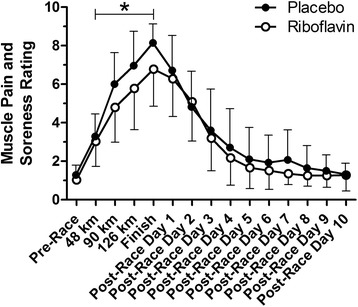
Mean lower-body muscle pain and soreness ratings for the 2 groups. *p = .043 for group comparison considering only the during-race and finish data. *Error bars* represent 1 SD and are shown only in 1 direction for clarity

The findings relative to muscle pain and soreness ratings for those subjects completing the study are shown in Fig. 1. Analysis of the data across all time points revealed no significant group (*p* = .14) or group by time interaction (*p* = .25), but there was a significant (*p* < .0001) time effect. Post-race values were statistically similar to pre-race values by post-race day 5. The pre-race and race data for all subjects providing data through 90 km (21 and 20 in riboflavin and placebo groups, respectively) were also analyzed. There was a significant group by time interaction (*p* = .023) and time (*p* < .0001) effect, but no group effect (*p* = .075), and post-testing revealed a significantly lower value (*p* < .01) for the riboflavin group than the placebo group at 90 km. This finding prompted further exploratory analysis of the data for those subjects completing the study with exclusion of the pre-race and post-race data (i.e., considering only the ratings during the race and at the finish) that yielded a significant (*p* = .043) group effect, as well as a significant (*p* < .0001) time effect, but no significant (*p* = .22) interaction effect.

**Fig. 2:**
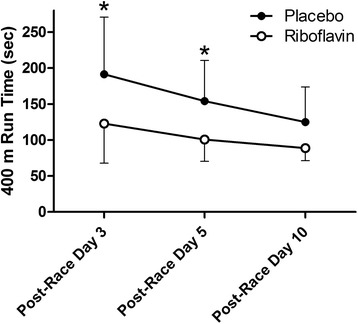
Mean 400-m run times for the 2 groups. *p < .05 for post-testing group comparison. *Error bars* represent 1 SD and are shown only in 1 direction for clarity

The post-race 400-m run times are shown in Fig. 2. Significant time (*p* < .0001) and group (*p* = .016) effects were found, but no significant (*p* = .60) interaction effect was evident. Post-testing revealed significant group differences (*p* < .05) at post-race days 3 and 5. For the 8 subjects who had completed 400-m runs prior to the race, the post-race day 10 times were not statistically different (*p* = .10) from the pre-race times (mean ± SD, 82 ± 9, 80 ± 10, and 85 ± 7 s for pre-race trial 1, pre-race trial 2, and post-race day 10, respectively).

As shown in Table 1, pain medication use during the race was not different between groups but was at 50% among the riboflavin group and 36% among the placebo group. Considering the 9 subjects in the riboflavin group who reported using pain medication during the race, 7 used a NSAID and 2 used acetaminophen at a mean (range) of 29% (12–62%) of the maximum recommended 24-h dose. Of the 6 subjects in the placebo group who reported using pain medication during the race, 1 used a NSAID and 4 used either acetaminophen, paracetamol, or paracetamol with codeine at a mean (range) of 39% (10–75%) of the maximum recommended 24-h dose. Comparison of muscle pain and soreness ratings between subjects who used pain medication during the race with those not using pain medication during the race revealed no suggestion of a group or interaction effect, whether considering all time points (*p* = .43 and *p* = .61 for group and interaction effects, respectively) or just the ratings during the race and at the finish (*p* = .98 and *p* = .82 for group and interaction effects, respectively).

Subjects who received riboflavin correctly suspected they had received the treatment 50% of the time, whereas 23% of the placebo group incorrectly thought they were in the treatment group. These rates of suspicion about being in the treatment group were not statistically different (*p* = .16). There were 3 subjects in the riboflavin group and 1 subject in the placebo group who noted a yellow urine color.

## Discussion

This work is a preliminary examination of riboflavin for potential benefits of reducing muscle pain and soreness during and after strenuous exercise and at enhancing recovery from strenuous exercise. The findings suggest that the vitamin may have some benefits. Indeed, riboflavin supplementation immediately before and midway during prolonged exercise appeared to be linked with reduced muscle pain and soreness during and at the completion of the exercise, and there was some evidence for enhanced functional performance during the initial several days after the exercise. While the findings require cautious interpretation, they are adequately interesting to warrant further investigation.

Given that post-race plasma CK concentrations were similar between groups, there is no evidence from this work that riboflavin acts by reducing muscle cell rupture. Rather, it would seem that it must act by altering the physiological response to exercise in other manners. While the underlying mechanism of action cannot be established from this study, it seems conceivable that the antioxidant properties of riboflavin [17–23] could explain reduced muscle pain and soreness during exercise, although the lack of reduced muscle pain and soreness during recovery does not seem consistent with this mechanism. On the other hand, the mitochondrial protective function of riboflavin [24] might be a plausible explanation for the riboflavin group demonstrating enhanced functional recovery without improvement in muscle pain and soreness during the recovery period.

Participants of the WSER are generally well-trained and experienced ultramarathon runners given that a recent qualifying ultramarathon is required to gain entry into the race. In this regard, they were conditioned for this type of activity and were adapted for controlling and responding to significant muscle injury from prolonged running. It is possible that an effect of riboflavin could be even greater in a group of subjects who are more naïve to strenuous exercise.

We chose to provide two 100-mg doses of riboflavin which were received immediately prior to exercise and around 11–16 h later during the approximately 20–30 h it took to complete the race. This dose and schedule were chosen because we felt it would be feasible to achieve subject cooperation and that any effectiveness should still be evident even if this was not the optimal dose or dosing schedule. The doses we provided were well above the recommended dietary allowance for riboflavin of 1.3 mg/day for adult men and 1.1 mg/day for adult women [41]. While the body absorbs little riboflavin from single doses beyond 27 mg [42], the vitamin appears safe at much higher doses [41] and riboflavin supplements are typically available in 100-mg capsules with recommendations to take 1–2 per day. Plasma concentrations of riboflavin and flavocoenzymes have been shown to peak around 2 and 3.5 h, respectively, after a single 60-mg dose of riboflavin, but the plasma concentrations remain elevated for hours [42]. Considering this information, it is possible that a lower dose at more frequent intervals might be more effective and would be recommended for future studies of this nature if feasible in the study environment.

The present findings indicate that muscle pain and soreness ratings of our subjects had returned to pre-race levels by 5 days after the race. For the subsample of 8 subjects who performed the pre-race 400-m runs, their times at 10 days after the race were statistically similar to pre-race times, although mean times were still ~5% slower at 10 days after the race. Our prior work at the WSER had also demonstrated that muscle pain and soreness ratings had statistically returned to baseline by post-race day 5, but 400-m run times were not examined in that study beyond post-race day 5 at which time pre-race performance had not been fully recovered [3]. In the present work, we extended the post-race time period of examination to 10 days and found that this appears to be close to the timeframe of 400-m run recovery. This is not intended to suggest that athletes are fully recovered from a 161-km ultramarathon within 10 days or shortly thereafter but rather that our subjective measure of resting muscle pain and soreness and our objective measure of 400-m speed had nearly recovered during this relatively short time period.

The WSER serves as an excellent environment to induce muscle pain and damage as evident from prior work at the race [2, 3, 5–7, 28–32]. This is confirmed with the present work in which muscle pain and soreness ratings at the end of the race averaged ~7–8 on the 10-point scale (sore to very sore) and median post-race plasma CK concentrations were ~7000–13,000 U/L. However, subject recruitment is a challenge in performing research of this nature in a competition setting such as the WSER. This is reflected in the number of subjects we were able to recruit. In particular, it is unfortunate that the number of subjects completing the pre-race 400-m runs was so low, which limits the robustness of our interpretation of the findings relative to functional recovery. While the treatment and placebo groups appeared to be well matched and blinding appeared to be adequate in this study, we cannot be certain that the groups were well matched for baseline performance at the 400-m run.

We acknowledge some other limitations with this study resulting largely from constraints related to the study being performed at a competition. Perhaps most importantly is that a sizable percentage of the subjects (50 and 36% in the riboflavin and placebo groups, respectively) used pain medication during the race and some used pain medication in the 10-day post-race period. The use of NSAIDs during this event has been common, ranging from 32 to 57% among those participating in our prior research [3, 34, 43]. Interestingly, earlier work has demonstrated that NSAID use during the race was not effective at controlling post-race muscle soreness [4] though the effect of NSAIDs on muscle pain and soreness during the race has not been systematically examined. Among the present subjects using pain medication during the race, the usual dosage was relatively low compared with the maximal recommended dose during 24 h. Not surprisingly, our separate comparison of muscle pain and soreness ratings between subjects who used pain medication during the race and those not using pain medication during the race revealed no suggestion of an effect of the pain medication on this variable. Thus, given these considerations, it seems unlikely that the use of pain medication during the race confounded the present finding of lower muscle pain and soreness during and at the completion of the race among the riboflavin group compared with the placebo group. Another potential study limitation is that, because we did not assess dietary practices of the subjects, it is conceivable that one group had a greater intake of anti-oxidants than the other. Additionally, the 400-m runs were unsupervised and self-timed, but this was the most feasible approach and we believe this study population was capable of maximally exerting themselves during unsupervised trials and correctly recording the times. Finally, we also recognize that some might consider it ideal to have measured pre-race plasma CK concentrations and to examine the pre-race to post-race change in plasma CK concentration rather than just the post-race value. But, since these runners would have reduced training prior to the race, we would expect pre-race plasma CK concentrations to have been very low relative to the post-race values, as previously demonstrated [4–7], so not using the pre-race to post-race change would not have altered our interpretation of the findings for this variable.

## Conclusions

From this work, we conclude that there is some evidence that riboflavin supplementation immediately before and midway through prolonged running may reduce muscle pain and soreness during and at the completion of the exercise and that there is some suggestion that riboflavin might enhance functional recovery after the exercise. We acknowledge that this study is a preliminary examination of riboflavin for this purpose and involved a small number of subjects in which the dose and dosing schedule might not have been optimal. As such, the findings appear intriguing and warrant additional investigation of riboflavin as a means to reduce muscle pain during exercise and to enhance post-exercise recovery.

## Abbreviations

ANOVA: Analysis of variance
CK: Creatine kinase
NSAID: Nonsteroidal anti-inflammatory drug
SD: Standard deviation
WSER: Western States Endurance Run
